# Distribution and risk assessment of microplastic pollution in a rural river system near a wastewater treatment plant, hydro-dam, and river confluence

**DOI:** 10.1038/s41598-024-56730-x

**Published:** 2024-03-12

**Authors:** Addrita Haque, Thomas M. Holsen, Abul B. M. Baki

**Affiliations:** Department of Civil and Environmental Engineering, Clarkson University, Potsdam, NY 13699 USA

**Keywords:** Freshwater ecology, Freshwater ecology, Environmental impact, Natural hazards

## Abstract

Rivers are the natural drainage system, transporting anthropogenic wastes and pollution, including microplastics (plastic < 5 mm). In a riverine system, microplastics can enter from different sources, and have spatial variance in concentration, physical and chemical properties, and imposed risk to the ecosystem. This pilot study presents an examination of microplastics in water and sediment samples using a single sample collection from the rural Raquette River, NY to evaluate a hypothesis that distinct locations of the river, such as downstream of a wastewater treatment plant, upstream of a hydro-dam, and river confluence, may be locations of higher microplastics concentration. In general, our results revealed the presence of high microplastic concentrations downstream of the wastewater treatment plant (in sediments), upstream of the hydro dam (both water and sediment), and in the river confluence (water sample), compared to other study sites. Moreover, the risk assessment indicates that even in a rural river with most of its drainage basin comprising forested and agricultural land, water, and sediment samples at all three locations are polluted with microplastics (pollution load index, PLI > 1; PLI_zone_ = 1.87 and 1.68 for water and sediment samples respectively), with risk categories between Levels I and IV (“minor” to “danger”). Overall, the river stands in a “considerable” risk category (PRI_zone_ = 134 and 113 for water and sediment samples respectively). The overall objective of this pilot study was to evaluate our hypothesis and advance our understanding of microplastic dynamics in rural river systems, elucidating their introduction from a point source (wastewater treatment plant), transit through an impediment (hydro-dam), and release into a vital transboundary river (confluence of Raquette-St. Lawrence Rivers).

## Introduction

Microplastics (plastics in the size range of 0.001–5 mm) have created a global concern due to their high concentration, ubiquitous presence, and harmful impact on the aquatic environment^1^. Over the years plastic products have been widely used for their hard-wearing, inexpensive, and lightweight properties. The popularity of plastic products can be understood from its rapid production growth. While plastic production was 1.7 million tons in 1950, in 2020 this number increased to 367 million tons and is predicted to reach 33 billion tons by 2050^2^. However, only 6–26% of plastics are reused, and a substantial volume of plastic waste is generated every year^3^. These plastic wastes gradually break down into small plastic particles due to anthropogenic and environmental influences or can be purposely manufactured as small plastic particles, both are known as microplastics.

Thompson et al.^4^ first introduced the concept of microplastics and in recent studies, microplastics have been found in inhospitable polar regions to the high-altitude land of the Himalayas^5^. Due to the small particle size microplastics can easily travel in all environmental compartments (atmosphere, water bodies, or soil). Transport and deposition of microplastics in water is dependent on the size and density of plastics^6^. Studies have also found that the smaller and less dense polymers remain suspended in water and are easily ingested by aquatic animals^7,8^. Bhutto and You^7^ also found that freshwater animals have certain preferences over the colors of ingestion; for example, white, yellow, and blue particles are more likely to be ingested. Peters et al.^9^ found that the shape of microplastics also plays an important role in the ingestion by aquatic animals; for example, the fiber type microplastics resemble worms and eggs and are often accidentally ingested by fishes. The human immune system and intestinal inflammation can be affected by these hazardous substances when entering the body through the food chain^10,11^. Wang et al.^12^ found that small particle size microplastics which have carcinogenic and mutagenic activity in humans can enter the blood, muscles, and liver tissues.

Although riverine systems deliver up to 80% plastic debris into the oceans, most research is focused on the marine pollution of microplastics^13^. Siegfried et al.^14^ found that 1.1 to 2.4 million tons of plastics are transported by rivers to the ocean and in the future decades this number could increase exponentially^15^. Riverine microplastics are mainly comprised of PE, PP, PVC, and PS^16^. The most common polymer type detected in rivers is polyethylene (PE) (42%), followed by polypropylene (PP) (30%), and polystyrene (PS) (11%). Other common polymer types like polyethylene terephthalate (PET), polyamide, and polyester are also often observed^17^.

As plastic production and waste are increasing, plastic pollution is becoming an environmental hazard^18^. According to the hazard level model of the United Nations Globally Harmonized System of Classification and Labelling of Chemicals (GHS), most plastic polymer types are grouped as hazardous substances^19^. Previously studies have been made on the abundance, properties, and toxicity of marine^20^, estuarine^21^, lacustrine^22^ and sediment^23^ microplastics, but not much progress has been made for freshwater environment risk of microplastics contamination, particularly in rural systems^24^. Therefore, urgent assessments of microplastic exposure in the environment and animals, along with analyses of contamination processes, are crucial for effective control measures of microplastics.

The river confluence is a very important part of the watershed in both hydrological and geomorphological aspects. In the confluence, currents from the two flows combine and result in an intensified turbulent mix of sediments and pollutants^22^. Also, at the confluence of rivers, suspended particle transport^25^, hydrodynamics, water quality, ecological patterns^26^, and riverbed morphology are altered significantly. More heterogeneous habitats are observed where effluents come together, which eventually leads to greater diversity and productivity in the confluences^27^. Therefore, confluences are regarded as biodiversity hotspots in fluvial networks^27^. Although a river confluence is a biodiversity hotspot in a fluvial network, the amount, type, and risk of microplastics entering from one river to another at a confluence has been understudied. Furthermore, studies on microplastics entering from a tributary of an important transboundary river are necessary to assess the ecosystem risk.

Wastewater treatment plants (WWTPs) are identified as a point source for microplastics entering into the aquatic and terrestrial environments, especially for synthetic fibers from clothing and plastics used in personal care products^28^. Even though wastewater is treated using both traditional and advanced treatment processes, a considerable amount of residual microplastics can still be present in the effluent^29,30^. Fibers and microbeads are the main types of microplastics found in wastewater treatment plants^31^. Globally, in a year, 360 km^3^ domestic and municipal wastewater is produced of which WWTPs treat 190 km^3^ (52.8%) and then discharge 149 km^3^^32^. Exfoliants and toothpaste release between 4500 and 95,500 and 4000 microbeads with each usage and about 35% of fiber microplastics are released to the oceans from synthetic textile washing^33^. A 5–6 kg weight of polyester and acrylic fabrics was observed to release 6,000,000 and 700,000 fibers, respectively^33,34^. The shear forces in the mixing or pumping machines of the wastewater treatment plant can further break microplastics into more and smaller particles resulting in a higher concentration of microplastics^35^. Therefore, microplastic studies on the upstream, effluent, and downstream of a WWTP is necessary to assess its microplastic contribution to the environment.

As an emerging contaminant in waterways, microplastic movement in rivers is gaining more attention. Obstruction of the natural movement of microplastics may result in significant changes in their concentration. For example, whether microplastics are accumulating behind dams has become an important question^36^. Dams limit the capacity of rivers to flow freely, altering many fundamental processes and activities that characterize healthy rivers^37^ and result in a fast decline in biodiversity and significant ecosystem services^38^. Dams are found to trap microplastics in reservoir sediments and the construction of dam may also alter the distribution of sediment microplastics^39^. In addition, as the river current velocity is significantly reduced upstream of a dam, different microplastic sampling techniques may be impacted differently causing additional uncertainty in the impacts of dams on microplastic transport and deposition.

In this pilot study, we tested our hypothesis that even in rural river system’s distinct riverine locations, such as a WWTP (considered a point source), a hydro dam (characterized as a flow impediment), and a river confluence (specifically, the Raquette-St. Lawrence River confluence) may serve as sources for microplastics hotspots by scrutinizing microplastic concentrations, size distribution, shape, color, polymer types. Using those results a risk assessment was performed. The specific objectives of this study were (1) to determine the change of microplastic abundance and properties near a wastewater treatment plant, hydro-dam, and a river confluence in a rural river system, (2) to compare different sampling techniques, including grab and net sampling for water samples and bed load and suspended sediment load for sediment load calculations, in determining microplastic concentrations; and (3) to conduct a thorough risk assessment to gauge the extent of microplastic pollution in the rural Raquette River.

## Materials and methods

### Research areas and sample collection

**Figure 1:**
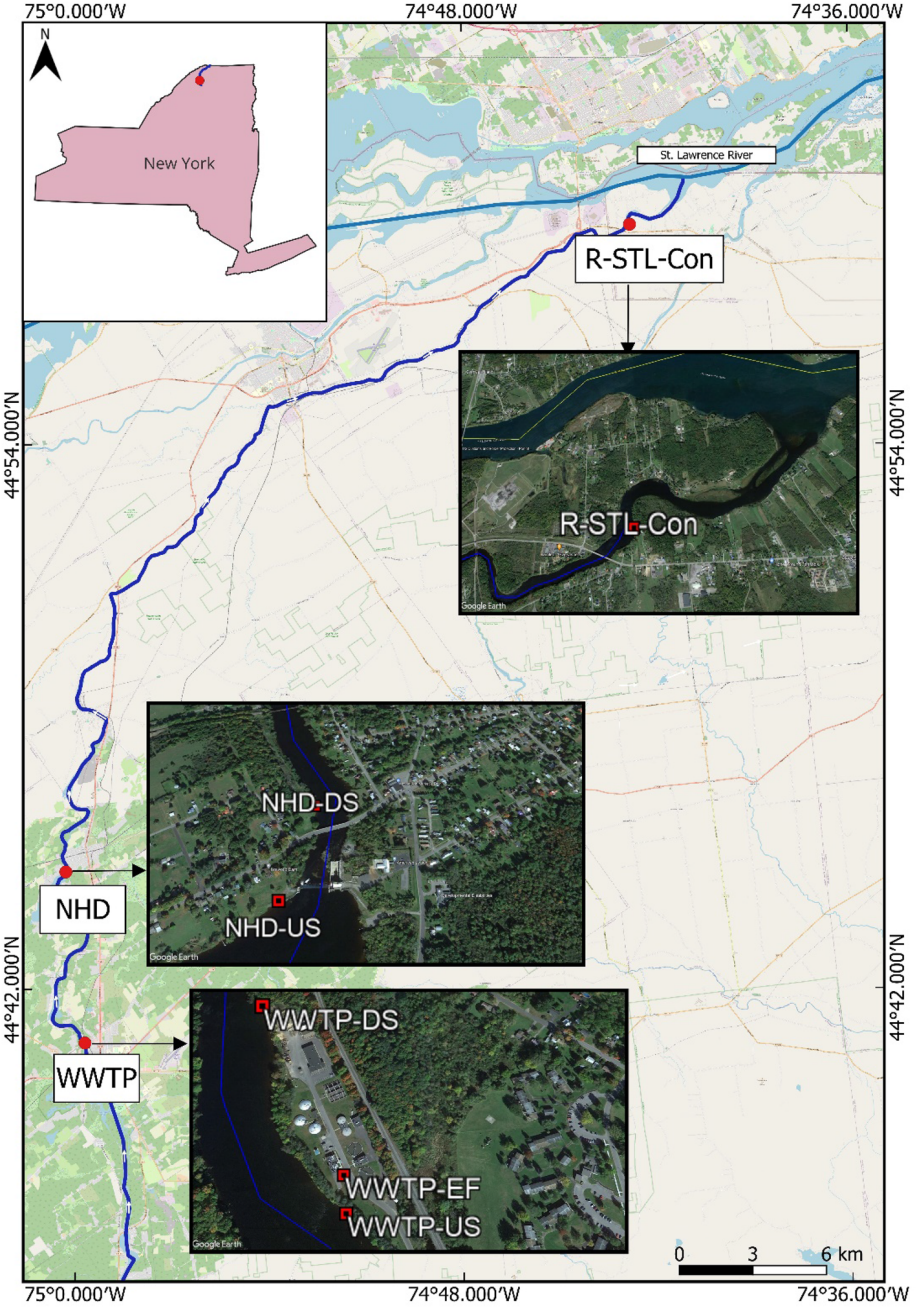
Map of Sampling Sites: Wastewater Treatment Plant (Upstream, Effluent and Downstream), Norwood Hydro-dam (Upstream and Downstream) and Raquette—St. Lawrence Rivers Confluence. Satellite imagery was obtained from Google Earth Pro. This map was created using QGIS Desktop 3.28.2 (https://www.qgis.org/en/site/forusers/download.html#). Shapefile of New York State was downloaded from NYS Civil Boundaries (https://gis.ny.gov/civil-boundaries).

For this pilot study, samples were collected from the Raquette River, Potsdam NY, an ecologically and culturally diverse regulated rural river system, that is a tributary of the St Lawrence River (Fig. 1). Originating from the Central Adirondack Region near Raquette Lake, New York, the Raquette River flows north approximately 280 km, drops more than 457 m in elevation to its confluence with the St. Lawrence River near Massena and creates a drainage basin of 2900 km^2^^40^. The river flows mostly in a sparsely populated region of private and public lands with limited and highly regulated development, extensive forest cover, and limited agricultural use^41^. The river's hydrology is very much influenced by rainfall and snowmelt and the river is also prone to seasonal flooding^42^. The highest river flow is during spring due to snowmelt, and lowest in the late summer and fall. Based on 62 years of from the USGS, the mean discharge of the Raquette river is 1220 cfs, minimum discharge was observed in 1967 (10 cfs) and maximum in 1982 (4340 cfs)^42^. Seventeen hydropower dams with 181 MW generating capacity were built in this river system^40^. These reservoirs also provide recreational opportunities like fishing, boating, and swimming. The Raquette River is well-known for its whitewater rapids and is a popular spot for kayaking and canoeing. Furthermore, the river supports local agriculture and is used for irrigation and water supply.

In this pilot study a single dataset of samples was collected from three different locations and six different sites of the Raquette River: upstream, effluent, and downstream of the Potsdam wastewater treatment plant (WWTP) outfall; upstream and downstream of the Norwood hydro-dam (NHD) and near the confluence of that Raquette River with St Lawrence River in Massena NY (Fig. 1, Table 1). The wastewater treatment plant upstream (WWTP-US) and downstream (WWTP-DS) sites were approximately 52 m upstream and 320 m downstream of the treatment plant. The Norwood hydro-dam upstream (NHD-US) and downstream (NHD-DS) sites were approximately 58.5 m upstream and 196.64 m downstream of the hydro-dam.

**Table 1:** Locations and sample collection time of the sampling sites.

Location	Sampling sites	Date	Time	Latitude	Longitude
Raquette-St. Lawrence confluence (R-STL-Con)	R-STL-Con	Sept 11, 2022	10:30 am	44° 58′ 51.80′′ N	74° 42′ 46.52′′ W
Wastewater Treatment Plant (WWTP)	WWTP-US	Oct 26, 2022	9:25 am	44° 40′ 49.13′′ N	74° 59′ 41.88′′ W
	WWTP-EF	Oct 26, 2022	9:30 am	44° 40′ 50.77′′ N	74° 59′ 42.19′′ W
	WWTP-DS	Oct 26, 2022	11:05 am	44° 41′ 0.37′′ N	74° 59′ 49.40′′ W
Norwood hydro-dam (NHD)	NHD-US	Sept 14, 2023	9:45 am	44° 44′ 35.38′′ N	75° 0′ 20.25′′ W
	NHD-DS	Sept 14, 2023	10:30 am	44° 44′ 39.96′′ N	75° 0′ 15.05′′ W

Where US, DS, and EF stands for upstream, downstream, and effluent sites of the sampling locations respectively.

For water sample collection, both a net (plankton net of mesh size 333 μm) and grab sampling were used. Before collecting a sample, plankton net was rinsed with river water three times. Then the net was suspended one foot below the water surface for five minutes facing upstream. Subsequently, the surface of the plankton net was rinsed with MilliQ water to gather the sample, and the resulting water was preserved in a glass bottle sealed with a metal cap. The time-averaged velocity (*V*) of the river flow was recorded using an Acoustic Doppler velocimetry (ADV) for at least two minutes before placing the plankton net in the water. The measuring point was approximately half a meter upstream of the plankton net entrance. From the obtained velocity, flow rate was determined using the continuity equation, *Q* = *A* * *V*; where *A* is the area of the plankton net entrance and *V* is the time-averaged velocity of the river flow. The volume of water (in liters, L) passing through the plankton net was then calculated using the equation, Volume = *Q* * *t*; where time, *t* = 5 min). Later, the number of microplastics per unit volume of water was determined (items/L). A metal water bucket was used to collect bulk water samples from the sites. Then three 1.0 L water samples were taken using a graduated cylinder and stored in a pre-cleaned glass jar. For sediment sample collection, both suspended sediment load and bed load were taken. Suspended sediment load can be defined as the particles that bounce along the channel, partly supported by the turbulence in the flow and partly by the bed. For collection of suspended sediment load, water behind an obstruction (e.g., stone or wood) was used to disturb the flow and then a 10 mL sample was collected using a turkey baster and stored in a 15 mL glass vial. For bed load, samples were collected from 0 to 5 cm of the riverbed and stored in a 15 mL glass vial before further treatment.

### Sample pretreatment

All three steps of laboratory processing (digestion, density separation and filtration) were carried out following the sample processing technique suggested by the National Oceanic and Atmospheric Administration (NOAA)^43^ with some modifications. First, water samples were digested using 20 mL 30% H_2_O_2_ with 20 mL 0.05 M Fe (II) solution at 75 °C for 30-min. An additional 20 mL of 30% H_2_O_2_ was added and then heated for 30 min more until no natural organic material was observed. Sediment samples were first dried at 70 °C for 24 h and then the oven dried weight was recorded. Next, samples were moved into a clean glass beaker and digested similarly as the water samples^44^. After digestion, samples were cooled to room temperature.

For density separation the most common salt used is NaCl as it is inexpensive and eco-friendly. However, because of the low density of NaCl (e.g., 1.2 g/cm^3^); NaI (density of 1.6–1.8 g/cm^3^) and ZnCl_2_ (density of 1.5–1.7 g/cm^3^) solutions are advantageous for the separation of high density polymers^45^. For this study, a concentrated saline solution (1.2 g/mL) of NaCl and (1.5 g/mL) ZnCl_2_ solutions were used to identify the low (PP, PE, PS) and high (PET, PVC) density polymers, respectively. The sodium chloride solution was prepared by dissolving 1.2 g/mL of NaCl in MilliQ water. The solution was added to the sample and manually stirred with a clean glass rod for 2 min. Then the concentrated ZnCl_2_ solution (1.5 g/mL) was added to the beaker and the supernatant was allowed to settle for 24 h. The beaker was covered with aluminum foil during this period. After density separation, the floating microplastics were vacuum filtered using a 0.45 µm glass fiber filter paper. Then, the filter papers were dried and covered with aluminum foil for a day at room temperature before microscopic analysis.

### Sample analysis

After extraction the microplastics were counted and photographed using a stereo microscope (Olympus SZX12, eyepiece 10X and maximum objectives 20X. Color camera LC30). When observing particles, the microscope was moved manually in a “zigzag” pattern from left to right until any microplastic was identified. This pattern continued thoroughly until the whole filter paper was examined. If a particle was identified as a microplastic, its image was captured and later analyzed for shape, size, and color using ImageJ. At least two of the following criteria were met to identify a particle as a microplastic to avoid misidentifying particles: (1) unnatural shapes e.g., perfectly spherical; (2) unnaturally brightly colored and a homogenous material or texture; (3) shiny/glassy; (4) without visible cells or organic structure; (5) without metallic luster; (6) with a uniform diameter and three-dimensional curvature (fiber morphology)^46^. After microscopic analysis, the microplastics were analyzed through Fourier Transformed Infrared Spectroscopy (FTIR) (Thermo Scientific, U.S.A) coupled with an Attenuated Total Reflectance (ATR) accessory^47^. The particles were scanned under the wavenumber from 500 to 4000 cm^−1^ using transmittance mode. The spectra were processed using OMNIC software (https://www.thermofisher.com/order/catalog/product/INQSOF018) with thermo scientific FTIR i10 and only polymers corresponding to reference spectra > 85% (compared to Open Specy—MicroplasticSpectra) were considered^48^.

### Quality control and quality assurance

All sampling equipment was precleaned with MilliQ water prior to every field trip. Water samples were stored in glass jars with metal lids and sediment samples were stored in glass vials. The laboratory bench was cleaned with filtered ethanol and only MilliQ water was used for rinsing the containers. To avoid artificial microplastic contamination and atmospheric transport, cotton laboratory aprons and nitrile gloves were worn throughout the laboratory processing. Not more than two individuals worked in the laboratory at the same time. Laboratory experiments were carried out under a laminar fume hood. In the laboratory, plastic stirrers were replaced with glass stirrers and after filtration, filter papers were covered immediately with aluminum foils. This sample processing technique was tested for the recovery of known microplastics (white polyethylene, WPMS = 1.10 g/cc, 150–180 µm, Cospheric) from MilliQ water and 97.4% recovery rate was found.

### Risk assessment of microplastics

While a standardized model for microplastic pollution has yet to be established, initial risk assessments are underway, considering their abundance, size, shape, and polymer type^49^. The risk assessment for the Raquette River was performed by adopting the methodology outlined in Xu et al.^50^. The pollution load index (PLI) of microplastics in the Raquette River was calculated using Eqs. (1) and (2) as detailed in Xu et al.^50^.

1 $$ PLI = C_{i} /C_{oi} $$

2 $$ PLI_{zone} = \sqrt[n]{{PLI_{1} \times PLI_{2} \times \cdots PLI_{n} }} $$

where, C_i_ is the abundance of microplastics at site i; and C_oi_ is the minimum abundance value for all sampling sites in the water and sediment samples of the Raquette River (water C_oi_ = 10 item/L, sediment C_oi_ = 110 item/kg (dry weight)) and PLI is the pollution load index of microplastics at a single sample point where *n* is the number of sampling points. PLI_Zone_ is the pollution load index of microplastics in the overall study area, and the degree of pollution is shown in Table 2.

**Table 2:** Pollution loading index (PLI), hazard index (H), and pollution risk index (PRI)^50^.

PLI		Risk Category	PRI	H	Risk Category
> 1	Polluted	Minor	< 150	< 10	I
		Medium	150–300	10–100	II
		Considerable	300–600	100–1000	III
		High	600–1200	1001–10,000	IV
		Danger	> 1200	> 10,000	V

The PLI risk assessment is based solely on the abundance of microplastics. However, this index does not provide a complete indication of risk as different microplastic polymers have different toxicity hazard score^19^. Hakanson^51^ proposed the idea of pollution risk index (PRI) that combines the PLI of microplastics and the hazard index of microplastic pollution (H) and therefore, evaluates the contamination level of microplastics in the environment. The pollution risk index (PRI) of the microplastic in the Raquette River was calculated using Eqs. (3)–(6).

3 $$ H_{i} = \Sigma P_{n} \times S_{n} $$

4 $$ H_{zone} = \sqrt[n]{{H_{1} \times H_{2} \times \cdots H_{n} }} $$

5 $$ PRI_{i} = H_{i} \times PLI_{i} $$

6 $$ PRI_{zone} = \sqrt[n]{{PRI_{1} \times PRI_{2} \times \cdots PRI_{n} }} $$

where *H*_*i*_ is the hazard index of microplastic pollution at site i, *P*_*n*_ is the mass fraction of different microplastic polymer types at the sampling site, *S*_*n*_ is the hazard score for microplastic polymers^19^, *H*_*Zone*_ is the hazard index of microplastic pollution of the Raquette River, *PRI*_*i*_ is the pollution risk index of microplastics at site i, and *PRI*_*Zone*_ is the pollution risk index of the overall area of the Raquette River.

### Statistical analysis

Microplastic concentration in each sample was calculated using the ratio between microplastic number and the total volume of water (*L*) for water samples and the ratio between microplastic number and dry weight of sediment (kg) for sediment samples^48^. Statistical differences in terms of microplastic concentrations between sampled locations or sampling method were tested using one-way ANOVA followed by a post-hoc Tukey test. *p*-values smaller than 0.05 were considered to indicate statistically significant differences. The statistical tests and graphs were performed using R Studio (v4.2.3, https://cran.r-project.org/src/base/R-4/) and Microsoft Excel (Version 2308, Build 16731.20170, https://learn.microsoft.com/en-us/officeupdates/current-channel#version-2308-august-28).

## Results and discussion

### Abundance of microplastics

*Sediment Samples* For sediment samples, both bed load and suspended sediment load were considered. Bed load samples were collected from the top 5 cm of the riverbed, thus the microplastics found may result from plastic accumulation over a longer time. On the other hand, suspended sediment load samples were collected using a turkey baster and disturbing the water behind an obstacle. For this reason, suspended sediment load represents recent deposits that are neither floating in the water column nor completely settling into the riverbed but remaining in suspended form. Significant variance was observed between microplastic abundances in suspended sediment load and bed load (F = 14.21, *p* = 0.0054) with the bed load having higher microplastic concentration.

**Figure 2:**
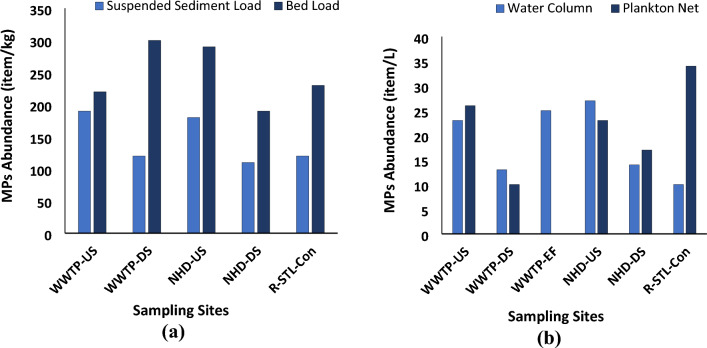
Microplastic abundance for: (**a**) sediment sample and (**b**) water sample.

The microplastic abundance in the sediment samples from three different locations of the Raquette River ranged from 110 to 300 items/kg (dry weight), with an average of 195 ± 67 items/kg (mean ± standard deviation) (Fig. 2a). In a previous study of the upper St. Lawrence River, the mean concentration of microplastics in the sediment sample was found to be somewhat higher than found here (832 $\pm 150$ items/kg dry weight)^52^. In Brisbane River, sediment samples collected over four different seasons had microplastic abundance in the range of 10–520 items/kg or 0.18–129.20 mg/kg^53^. In other similar studies the microplastic abundance in urban freshwater streams in Adelaide, Australia was 6.4 ± 5.5 particles/L^54^ and the surface water of Awano River, Ayaragi River, Asa River and Majime River of Japan, was found to be 132.80 ± 15.3 items/L, 111.88 ± 21.42 items/L, 130 ± 27.84 items/L and 272.50 ± 299.15 items/L, respectively^55^; and microplastic abundance in sediment load was found to be 288 ± 60 items/kg in Xiangjiang river, China^56^; 590 particles/kg in Jagir Estuary, Indonesia and 31 particles/kg in estuary, UK^57^. Overall, the average microplastic concentration in the Raquette River (20.2 ± 7.86 items/L for water sample and 195 ± 67 items/kg for sediment sample) was found to be lower than other freshwater samples reported in the reviewed literature, except for the urban freshwater streams in Adelaide, Australia and in the sediments of Jagir Estuary, Indonesia.

At the Potsdam wastewater treatment plant site, the average microplastic concentration in upstream and downstream sediment samples were 205 and 210 items/kg respectively. The relatively higher microplastic concentration in downstream sediment samples could be due to MP present in the effluent discharge from the wastewater treatment plant and geometry of the river cross section (i.e., pool). At the Norwood hydro-dam site, the average microplastic concentration in upstream and downstream sediment samples were 235 and 150 items/kg respectively. The increase in microplastic concentration in dam upstream sites suggests that dams act as a barrier to microplastic movements and therefore, microplastics tend to settle in the upstream region. The average microplastic concentration in the Raquette-St. Lawrence confluence site was 175 items/kg, which did not vary significantly from the other two locations.

Results from this study represent microplastic abundance for a short period of time. We posit a hypothesis suggesting that the variability in microplastic concentration within river sediment exhibits greater prominence along the vertical axis compared to the longitudinal axis. We recommend the exploration of diverse scenarios, incorporating alterations in river flow rates and accounting for seasonal influences for future investigations in this domain.

*Water Samples* Both plankton net and grab sampling methods were used in this pilot study. The plankton net, submerged in the river for a duration of five minutes, provided a somewhat integrated sample, while the grab sample was collected instantaneously. The plankton net, submerged in the river for a duration of five minutes, provided an integrated sample over that time interval providing a more comprehensive representation of microplastics because if sampled a larger water volume. In contrast, the grab sample method generates data for a single point in time for a smaller water volume. Therefore, the plankton net sampling method is advantageous for assessing microplastic content in larger water volumes, while bulk sampling provides a snapshot of microplastic status in a smaller, specific volume of water at a particular point of time. Further studies need to analyze the advantages and disadvantages of grab and net sampling techniques for better understanding of the strengths and limitations of each method. Analysis revealed that microplastic abundance derived from plankton net sampling at the confluence of the Raquette and St. Lawrence rivers was nearly three times that of grab sampling (34 and 10 items/L, respectively). However, microplastic abundance did not exhibit significant variation based on sampling techniques in water samples collected from the wastewater treatment plant and Norwood hydro-dam (F = 0.46, *p* = 0.51). Our results suggest that the choice of sampling technique may hold important contingent upon the specific sampling location within a river, necessitating validation of results through multiple sampling methods. Microplastic concentration found for grab and net sampling in wastewater treatment plant upstream (WWTP-US) was 23 and 26 items/L, in wastewater treatment plant downstream (WWTP-DS) was 13 and 10 items/L, in Norwood hydro-dam upstream (NHD-US) was 27 and 23 items/L, and in hydro-dam downstream (NHD-DS) was 14 and 17 items/L respectively. In the rest of the sections average of net and grab sampling concentration would be reported for microplastic concentration in the water sample.

The average microplastic concentration found in the water sample of Raquette river was 20.2 ± 7.86 items/L (mean ± standard deviation) (Fig. 2b). The minimum observed microplastic concentration was 10 items/L, surpassing the established threshold value for food dilution effects, determined through organismal and population scale toxicity tests (5 microplastics/L), as outlined in the 2024 California Integrated Report on surface water quality assessments^52^. The maximum microplastic concentration was identified at the Raquette-St. Lawrence confluence (34 items/L), aligning with previous research indicating greater microplastic concentrations in confluence areas compared to upstream regions^48^. High concentration of microplastics in the confluence area has also been reported for Cisadane River (32 MP/m^3^), Ottawa River (120 MP/m^3^), Milwaukee River (1.27 MP/m^3^), Wei River (950 MP/m^3^), and Mekong River (3.96 MP/m^3^) respectively^55–59^. Notably, the Raquette-St. Lawrence confluence site was also the most downstream location within the scope of this study (approximately 53.4 km downstream from the Potsdam wastewater treatment plant) and also is situated downstream of the town of Massena, which has a higher population density than the upstream region, the village of Potsdam (2234/sq mi and 1679/sq mi respectively)^60,61^. This observation supports our hypothesis that microplastic concentration has a positive correlation with location (the most downstream site of a study area may have maximum microplastic concentration if deposition is slow relative to transport), population density of the respective area (higher population density may contribute in high microplastic abundance in the river), river dynamics, and sampling technique (sampling technique considering river velocity (e.g., net sampling) may provide higher microplastic concentration than grab sampling).

The Potsdam wastewater treatment plant, with a nominal treatment capacity of 5 million gallons per day, typically processes an average daily volume ranging from 1 to 1.2 million gallons^62^. Microplastic concentrations were assessed at three distinct locations within the wastewater treatment system, designated as upstream (WWTP-US), effluent (WWTP-EF), and downstream (WWTP-DS). Results revealed average microplastic concentrations of 24.5, 25, and 11.5 items per liter (items/L) at WWTP-US, WWTP-EF, and WWTP-DS, respectively. The upstream and downstream samples were collected near the riverbank and the effluent sample was collected from the effluent tank of the treatment plant. Notably, the upstream and downstream samples were procured 52 m upstream and 320 m downstream of the wastewater treatment plant, respectively. For the wastewater treatment plant site, part of our hypothesis is true that microplastic concentration would be a maximum in the effluent impacted site. Even though this was not observed in the water samples it is imperative to interpret these results as a temporal snapshot, acknowledging the dynamic nature of microplastic concentrations within wastewater treatment plant effluents. The decrease in downstream microplastic concentration might have been due to the sedimentation of microplastics or that the downstream samples were not indicative of the mean concentration. We posit that multiple factors, including but not limited to distance, sample collection time, geometry of the river cross section, and effluent release timing, should be systematically considered, and reported when assessing microplastic concentrations downstream of wastewater treatment plants. This hypothesis underscores the nuanced and multifaceted nature of the dynamics governing microplastic distribution in aquatic environments influenced by wastewater treatment plant discharges.

The Norwood hydro-dam is an active hydro project of total capacity of 2 MW from hydraulic turbine-generator units and an annual net hydropower generation of 12,493 MWH^63^. For Norwood hydro-dam, the average microplastic concentration in upstream and downstream sites were 25 items/L and 15.5 items/L respectively. This result supports our hypothesis that obstructions in a natural river, e.g., dam, traps the microplastics and results in a high concentration of microplastics in the upstream region. This observation matches findings from prior studies where dams and reservoirs were found to be a sink for microplastics^62^. Microplastic at the dam site could enter from various sources, such as-plastic litter thrown by the visitors coming to dam, domestic waste from the nearby households, particles from the dam operating site (e.g., fragments of weirs, cables, equipment or construction and maintenance materials), fragments from the kayaking and canoeing boat, etc.

### Size distribution

**Figure 3:**
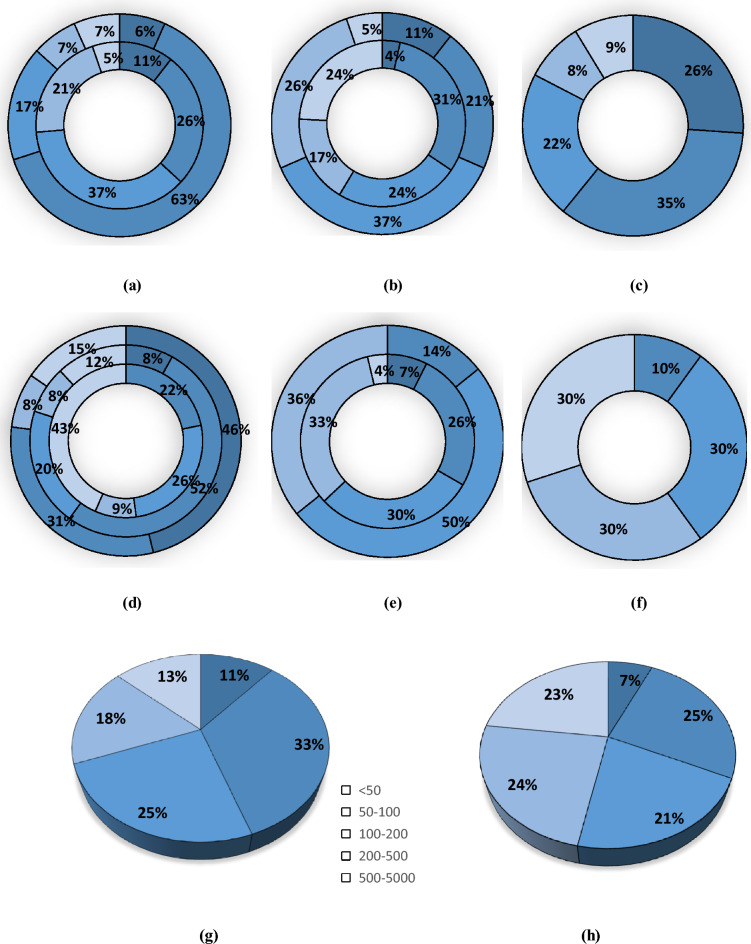
Percentage of microplastics sizes at different sampling locations in sediment samples: (**a**) WWTP, (**b**) NHD, (**c**) R-STL-Con; in water samples: (**d**) WWTP, (**e**) NHD, (**f**) R-STL-Con; overall microplastic percentage in (**g**) sediment sample, (**h**) water sample. For WWTP site (a & d), the inner circle represents WWTP-US, middle circle represents WWTP-EF and outer circle represents WWTP-DS. For NHD site (b & e), the inner circle represents NHD-US and outer circle represents NHD-DS.

Microplastics were classified in five size categories: < 50, 50–100, 100–200, 200–500, and 500–5000 µm (Fig. 3). No single size class dominated the water and sediment samples. In water samples, the most abundant size class was 50–100 µm (25%), followed by 200–500 (24%), 500–5000 (23%), 100–200 (21%) and < 50 µm (7%). In sediment samples, the 50–100 µm (33%), was also the most abundant size class followed by 100–200 (25%), 200–500 (18%), 500–5000 (13%) and < 50 µm (11%). It was observed that the smaller three size classes (i.e., < 50, 50–100 and 100–200 µm) were more abundant in sediment samples (11%, 33% and 25% for sediment samples and 7%, 25% and 21% for water samples respectively) and the larger two size classes of microplastics (200–500 and 500–5000 µm) were more abundant in water sample (21%, 23% for water sample, and 18%, 13% for sediment samples respectively). In literature, microplastics of different size classes were also observed for water and sediment samples. For example, dominant microplastic sizes for both water and sediment samples of West River downstream, in the south of China was < 0.5 mm^63^; for sediment samples of Brisbane River, Australia, this value was < 3 mm^64^ and in the sediments of Karnaphuli River Estuary, Bangladesh, it was 1–5 mm (30.38%).

The destruction of the structural integrity of the plastic under conditions of external heat, weathering, or shearing of current wave or UV radiation, polymers might have formed irregular shapes and sizes. Therefore, more fragmented particles were found in greater number for the sediment sample where the microplastics are accumulated for long time and less fragmented particles were found in greater number for the water sample where microplastics are mostly floating. In the water sample of the wastewater treatment plant, the upstream site was highly concentrated with 500–5000 µm microplastics (43%), effluent with 50–100 µm microplastics (52%), and downstream with < 50 µm microplastics (46%) which might be due to the deposition of larger microplastic in the upstream, resulting in a high concentration of smaller microplastics in downstream. Similar observations could be made for the sediment samples as well as the smaller microplastic size are more abundant in downstream site (50–100 µm in 63%) than upstream site (100–200 µm in 37%).

At the Norwood hydro-dam, microplastics of all five size classes were found in the upstream water sample and the dominant three size classes were: 200–500 µm (33%), 100–200 µm (30%) and 50–100 µm (26%). However, microplastics of only three size classes were found in the downstream of the dam with the highest abundance of 50% for 100–200 µm (50%) followed by 200–500 (36%) and 50–100 µm (14%). From this size distribution of the upstream and downstream water sample of a dam, it can be inferred that the smallest and largest size class of microplastics might be entrapped in the upstream water and sediment samples of the dam or may start accumulating in the downstream sediment sample and therefore, remain absent in the downstream water sample. In the sediment sample, the missing size classes from the downstream water sample (< 50 µm and 500–5000 µm) were found in both upstream (4% and 24% respectively) and downstream (11% and 5% respectively) of the dam.

From the size class percentages of the upstream and downstream sediment samples it can be assumed that the upstream sediment sample tends to entrap the larger size microplastics whereas the downstream sediment sample accumulates the smaller size microplastics. The high velocity of water current in the dam downstream site might be the reason for fragmentation of the microplastics and the low velocity of water in the upstream site might result in entrapment of larger microplastics.

The R-STL-Con site, water sample showed four size classes of microplastics (100–200 µm, 200–500 µm and 500–5000 µm each 30% and 50–100 µm in 10%). Since this was the most downstream site of this study, it can be assumed that microplastics from different sources get mixed in this site resulting in an almost homogenous size class. The sediment sample of this site was abundant in all five classes and the highest abundance was observed for 50–100 (35%) µm, followed by < 50 µm (26%) and 100–200 µm (22%). This result again proves that the most downstream site (river confluence) of this study is enriched with different sizes of microplastics but with a tendency of accumulating smaller microplastics in the sediment sample.

### Shape distribution

**Figure 4:**
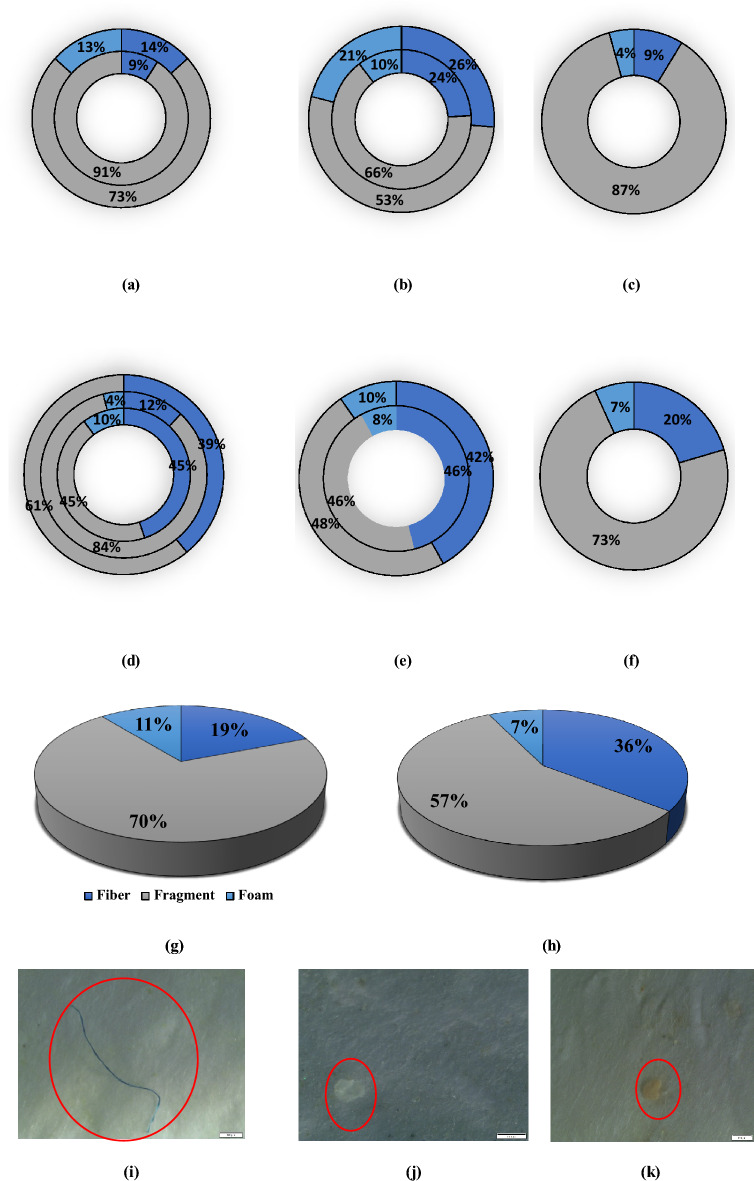
Percentage of microplastics shapes at different sampling points in sediment samples: (**a**) WWTP, (**b**) NHD, (**c**) R-STL-Con; in water samples: (**d**) WWTP, (**e**) NHD, (**f**) R-STL-Con; overall microplastic percentage in (**g**) sediment sample, and (**h**) water sample. Microplastics shape observed in this study: (**i**) fiber, (**j**) fragments, (**k**) foam. For WWTP site (a & d), the inner circle represents WWTP-US, middle circle represents WWTP-EF and outer circle represents WWTP-DS. For NHD site (b & e), the inner circle represents NHD-US and outer circle represents NHD-DS.

Microplastics were classified into three types in terms of shape (fibers, fragments, or foams) (Fig. 4). Fragment type microplastics were found to have the highest percentage for all water and sediment samples, ranging from 45 to 84% for water samples and 53% to 91% for sediment samples. Most (57%) of all water samples were fragments, followed by fiber (36%) and foam (7%). For sediment samples, 70% of total microplastics were fragment followed by fiber (19%) and foam (11%). Similar findings have been observed in the recent literature for river water and sediment samples, as in both cases the dominant microplastic types are fiber and fragments.

Although the morphological distribution of the microplastics in the water and sediment samples were similar, fragment concentrations were higher at every site for the sediment samples. Min et al.^49^ hypothesized that under the hydraulic action fragmented microplastics would be washed to the bank with sediment and therefore, sediment samples would be richer in fragments than the water samples. Wastewater treatment plants are considered to have limited effectiveness in removing fiber type microplastics. Therefore, downstream of the wastewater treatment plant though fibers have decreased in the water sample (45% to 39%), they increased for sediment sample (9% to 14%). At the Norwood hydro dam site neither fragments nor fibers were more prevalent. However, foam type microplastics were found to be as high as 21% in sediment sample of the NHD-DS site. This could be the result of surface runoff from the paper mill (Potsdam Specialty Paper, Inc), situated upstream of the hydro dam. The high concentration of fragments in both water (73%) and sediment samples (87%) at the R-STL-Con site is as expected as the river flows through several roads, automobile facilities like car wash, towing or garage, parks, restaurants, households, and stormwater runoff can easily pollute the river. Particles from the car tire, plastic bottles or bags might have contributed to high fragment concentration in this area.

### Color distribution

**Figure 5:**
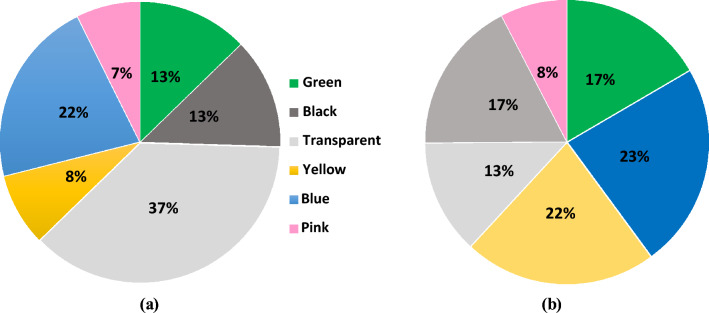
Microplastics colors in the samples: (**a**) sediment and (**b**) water.

The color classification of microplastics in the water and sediment samples of the Raquette River is shown in (Fig. 5). Microplastics of six different color types were found in this study area: green, black, transparent, blue, yellow, and pink. No color is dominant in either water or sediment sample and all these colors represent plastic products used in daily commodities. For example, domestic microplastics consist of colorful fibers from clothes, fragments from broken plastic containers or torn plastic bags. Furthermore, the fragments of car tires and trash bags might have increased the percentage of black microplastics in this area. Additionally, fragments from the disposable lunch boxes, plastics bags or cup lids might have been transported by surface runoff and contributed as a source of transparent microplastics for water and sediment samples. Moreover, colorful microplastics may also fade and turn transparent due to ultraviolet irradiation, water erosion or activities of microorganisms^49^.

### Composition of microplastics

**Figure 6:**
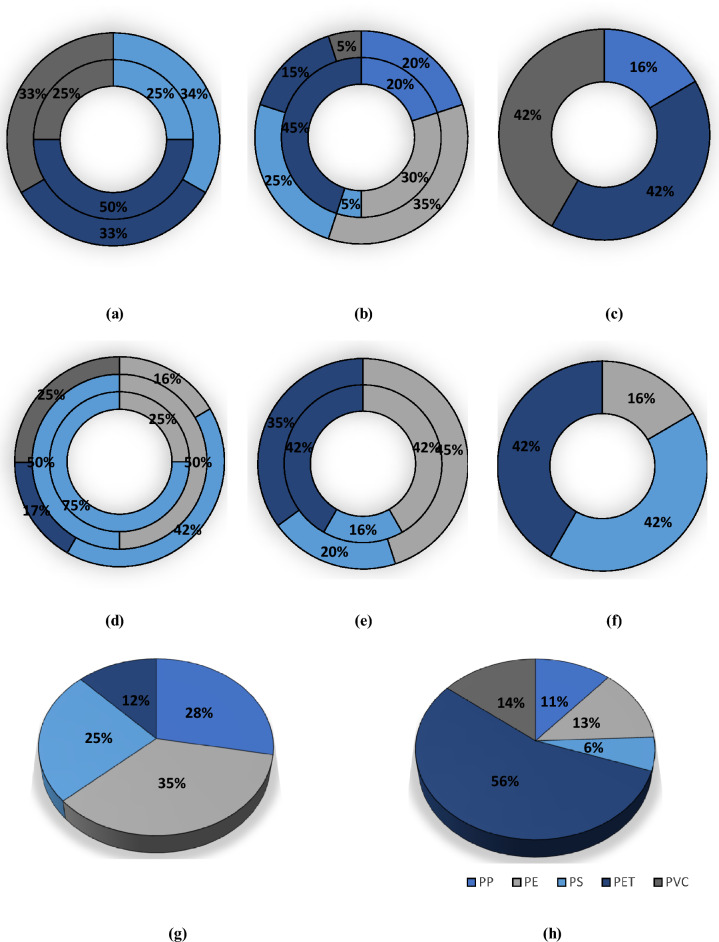
Percentage of microplastics polymer types at different sampling points in sediment samples: (**a**) WWTP, (**b**) NHD, (**c**) R-STL-Con; in water samples: (**d**) WWTP, (**e**) NHD, (**f**) R-STL-Con; overall microplastic percentage in (**g**) sediment sample, (**h**) water sample. For WWTP site (a & d), the inner circle represents WWTP-US, middle circle represents WWTP-EF and outer circle represents WWTP-DS. For NHD site (b & e), the inner circle represents NHD-US and outer circle represents NHD-DS.

The microplastics were classified into five categories: polyethylene terephthalate (PET), polystyrene (PS), polypropylene (PP), polyethylene (PE), and polyvinyl chloride (PVC) (Fig. 6). In the water sample, polymers of PE, PP, PS and PET were detected but in the sediment samples all five polymer types were present. The dominant microplastic in water samples was PE (35%), followed by PP (28%), PS (25%) and PET (12%). For sediment samples, the dominant microplastic was PET (56%), followed by PVC (14%), PE (13%), PP (11%) and PS (6%). The high-density polymers (PET and PVC, density > 1 g/cc) are more abundant in sediment samples and the low-density polymers (PE, PP, and PS, density < 1 g/cc) are more abundant in water samples. This result indicates that the high-density plastics are getting deposited where the low-density plastics are staying afloat in the environment.

PE and PET are the components of the containers used in our daily life (e.g., high-density, juice bottles, shampoo/ conditioner bottles or food containers). High density polyethylene (HDPE) is used in the making of trash bags, pipes and pipe fittings or ropes for its high strength and durability. Most of the raw materials for textile products in daily life (e.g., clothing, blankets, etc.) contain PET. Also, in the process of washing and using such items large amounts of PET fibers are released^49^. PS is widely used in protecting consumer products, in the form of solid and foam (e.g., egg cartons, foam packaging of peanuts for shipping, food packaging, meat/ poultry packaging or in CD/DVD cases).

As WWTP discharges effluents from the domestic wastes, high concentration of PE, PS or PET were observed. Furthermore, the NHD site is also polluted by domestic waste as well as litter from the visitors to the dam. Therefore, similar microplastics type is also found for this site. PP is one of the most widely used thermoplastics in the world and therefore used in car parts, housewares, toys, and packaging. PVC is another well-known high strength thermoplastics, widely used for pipes, wires and cable insulation. Surface runoff from automobile facilities (e.g., car wash, garage, car towing, and tire shops), household effluents, recreational parks and roads might have contributed to PP and PVC concentration for the most downstream site, R-STL-Con. Similar findings have been observed in recent literature on riverine microplastics. The dominant microplastic type in the surface water of Majime River, Japan, was PP, PS and PET; in the sediment sample of Brisbane River, PE, PA, PP and PET were the most abundant polymer type; in surface water and sediment samples of West River downstream, PP, PE, PET, PVC and PET were majority^64^.

### Preliminary risk assessment of a rural river

**Figure 7:**
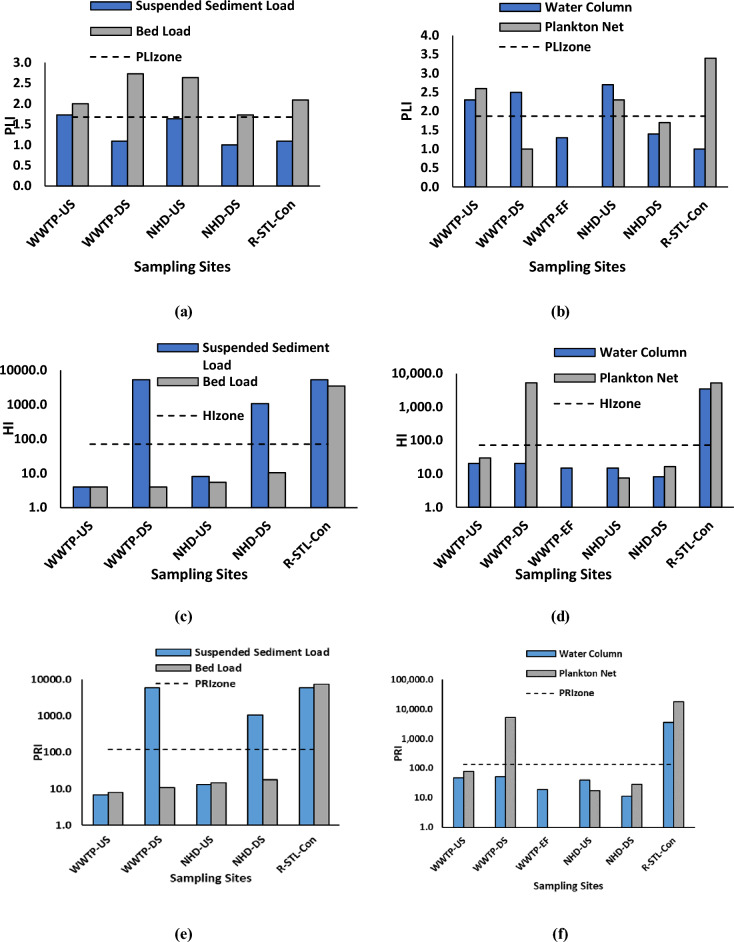
Ecological risk assessment of MPs pollution in the Raquette River: (**a**) PLI of sediment sample (**b**) PLI of water sample, (**c**) HI of sediment sample, (**d**) HI of water sample, (**e**) PRI of sediment sample, and (**f**) PRI of water sample.

To discuss the impact of microplastics in the rural Raquette River, three risk indices: pollution load index (PLI), hazard index (HI), and pollution risk index (PRI) were calculated (Fig. 7). The highest PLI value for water and sediment samples were 3.40 and 2.72 for R-STL-Con PN sample and WWTP-DS BL sample respectively, indicating that microplastic pollution is highest in a river confluence for water samples and downstream of a wastewater treatment plant for sediment samples As the Raquette River flows from upstream to downstream, it passes several microplastic sources, and therefore reaches the highest microplastics concentration at the most downstream site, resulting in the highest PLI among the water samples.

For sediment samples, sediment downstream of wastewater treatment plant is directly exposed to the wastewater effluents and thus becomes enriched in microplastics. Furthermore, comparing the PLI_zone_ = 1.87 for water sample with the PLI_zone_ = 1.68 for sediment sample, it can be referred that water samples are more polluted than sediment samples. However, the ecological risk of microplastics depend on both the concentration of microplastics as well as the toxicity of the chemicals present in the microplastics according to the GHS hazard level model as different polymer has different toxicity hazard scores (e.g., hazard score of PP and PVC is 1 and 10,001 respectively). Since different polymers can harm the environment to different degrees, they receive different hazard scores.

The H values of water samples ranged from 7.50 to 5290 for water samples and 4 to 5277 for sediment samples. Hazard Index (HI) < 10 represents risk category I, for risk category II, III, IV, and V, this range is 10–100, 100–1000, 1001–10,000 and > 10,000 respectively^50^. For water sample, NHD-US PN and NHD-DS WC samples are in risk category I (HI = 7.50 and 8.20 respectively); WWTP-DS WC, NHD-US WC, NHD-DS PN, WWTP-US WC, WWTP-EF and WWTP-US PN are in risk category II (HI = 14.85, 14.85, 16.50, 20.50, 20.50, and 30 respectively); R-STL-Con WC, R-STL-Con PN and WWTP-DS PN are in risk category IV (HI = 3493, 5277 and 5290 respectively). Similarly, for sediment, WWTP-US SSL, WWTP-US BL, WWTP-DS BL, NHD-US BL and NHD-US SSL samples are in risk category I (HI = 4, 4, 4, 5.50, and 8.10 respectively); NHD-DS BL is in risk category II (HI = 10.40); NHD-DS SSL, R-STL-Con BL, WWTP-DS SSL and R-STL-Con SSL are in risk category IV (HI = 1069, 3483, 5277 and 5277 respectively). Samples with higher HI value contain PVC and PS. Also, PLI and HI vary significantly among the sites for water sample (F = 3.67, *p* = 0.069) but do not vary significantly for sediment sample (F = 4.47, *p* = 0.048). H_zone_ of microplastics for water and sediment samples is 72.13 and 70.60 respectively both lies in risk category III. Since the HI value of microplastic is significantly related to the toxicity score of polymers, use and production of high toxic polymer should be reduced.

Finally, the PRI of the Raquette River was calculated by combining PLI and H^51^. Pollution Risk Index (PRI) < 150 represents minor risk category; for medium, considerable, high, and danger risk category this range is 150–300, 300–600, 600–1200 and > 1200 respectively^50^. For water sample, R-STL-Con WC, WWTP-DS PN and R-STL-Con PN samples are in “danger” risk category (PRI = 3493, 5290, and 17,943 respectively) and rest of the samples fall in “minor” risk category. For sediment sample, WWTP-DS SSL, R-STL-Con SSL, R-STL-Con BL fall in danger risk category (PRI = 5757, 5757, and 7283); NHD-DS SSL fall in high-risk category (PRI = 1069) and rest of the samples are in minor risk category. PRI_zone_ of microplastics for water and sediment samples is 134 and 118 respectively both lies in “Considerable” risk category. It is important to note that both the water and sediment samples of the R-STL-Con sites possess very high ecological risk. The presence of several potential sources of microplastics in the environment has severely polluted the confluence area. Furthermore, downstream of both wastewater treatment plant and the hydro-dam have showed significant rise in the ecological risk from the upstream site. Therefore, it is advised to ensure proper treatment of the high-risk polymer before discharging into the environment.

## Conclusion

This pilot study involved a brief investigation of microplastic pollution of the Raquette River at three distinct sites: the Potsdam wastewater treatment plant (WWTP), the Norwood hydro-dam (NHD), and the Raquette-St. Lawrence River confluence (R-STL-Con). The general findings reveal that these sites not only influence the presence and types of microplastics but also bring changes in the ecological risk assessment in the river system. From 11 water samples and 10 sediment samples, the maximum concentration of microplastics was found in R-STL-Con site and WWTP-DS site for water (34 items/L) and sediment (300 items/kg) sample respectively, which signifies that the presence of potential sources along the river increases the microplastic concentration in water sample but for sediment sample high concentration of microplastics is more likely to be found close to the source. Furthermore, the presence of Norwood hydro-dam has contributed in increasing microplastic concentration in the upstream site, which implies that the presence of an obstacle significantly impacts the movement of microplastics. Therefore, our hypothesis was partially proved that downstream of a wastewater treatment plant (WWTP), upstream of a hydro dam, and river confluence have higher microplastics concentration for a river system. The color, size, shape, and polymer type of the microplastics found in this study indicates that these plastics are coming from the daily used products. Moreover, even in a rural river system the presence of microplastics poses a high ecological risk for the environment. For future research, it is encouraged to test the hypothesis of this study in changing environment scenarios (e.g., flow rate, seasonal impact, rainfall events, etc.). The general observation from this study is that to reduce the amount as well as ecological risk of microplastics in environment, with proper treatment and policy making, reduction of plastic product should be adopted in our daily life.

## Data Availability

The datasets generated during and/or analyzed during the current study are available from the corresponding author on reasonable request.
